# Novel Biomarkers in Early Detection of Heart Failure: A Narrative Review

**DOI:** 10.7759/cureus.53445

**Published:** 2024-02-02

**Authors:** Maryam Kayani, Neha Fatima, Pooja Chowdary Yarra, Naiela E Almansouri, Deepshikha K, Abirami Balasubramanian, Navya Parvathaneni, Adetola G Mowo-wale, Josue A Valdez, Zahra Nazir

**Affiliations:** 1 Cardiology, Shifa Tameer-e-Millat University Shifa College of Medicine, Islamabad, PAK; 2 Internal Medicine, Lisie Hospital, Kochi, IND; 3 Internal Medicine, American International Medical University, Gros islet, LCA; 4 Internal Medicine, California Institute of Behavioral Neurosciences & Psychology, Fairfield, USA; 5 Internal Medicine, University of Tripoli, Tripoli, LBY; 6 Cardiology, Pondicherry Institute of Medical Sciences, Pondicherry, IND; 7 Medicine, Stanley Medical College, Chennai, IND; 8 Public Health, Sacred Heart University, Connecticut, USA; 9 Internal Medicine, Obafemi Awolowo College of Health Sciences, Sagamu, NGA; 10 General Practice, Universidad Autónoma de Durango, Los Mochis, MEX; 11 Internal Medicine, Combined Military Hospital, Quetta, PAK

**Keywords:** procalcitonin, cardiac troponin, bnp, serum biomarkers, heart failure

## Abstract

Heart failure (HF) represents a significant global health challenge, characterized by a variety of symptoms resulting from cardiac dysfunction. This dysfunction often leads to systemic and pulmonary congestion. The pathophysiology of HF is complex, involving stimulation of the sympathetic nervous system, which is insufficiently balanced by the release of natriuretic peptide. This imbalance leads to progressive hypertrophy and dilatation of the heart’s chambers, impairing its pumping efficiency and increasing the risk of arrhythmias and conduction disorders. The prevalence of HF is exceptionally high in industrialized nations and is expected to increase owing to an aging population and advancements in diagnostic methods. This study emphasizes the critical role of early diagnosis in reducing morbidity and mortality associated with HF, focusing specifically on the evolving importance of biomarkers in managing this condition.

Biomarkers have played a key role in transforming the diagnosis and treatment of HF. Traditional biomarkers such as b-type natriuretic peptide and N-terminal pro-b-type natriuretic peptide have been widely adopted for their cost-effectiveness and ease of access. However, the rise of novel biomarkers such as growth differentiation factor 15 and adrenomedullin has shown promising results, offering superior sensitivity and specificity. These new biomarkers enhance diagnostic accuracy, risk stratification, and prognostic evaluation in HF patients. Despite these advancements, challenges remain, such as limited availability, high costs, and the need for further validation in diverse patient populations. Through a comprehensive literature review across databases such as PubMed, Google Scholar, and the Cochrane Library, this study compiles and analyzes data from 18 relevant studies, offering a detailed understanding of the current state of HF biomarkers. The study examines both traditional and emerging biomarkers such as galectin-3 and soluble suppression of tumorigenicity 2 in HF, exploring their clinical roles and impact on patient outcomes.

## Introduction and background

Globally, heart disease is one of the most significant contributors to morbidity and mortality. According to the World Health Organization (WHO), cardiovascular diseases (CVDs) cause 17.9 million deaths annually, or 31% of all fatalities worldwide. Improving patient outcomes with heart disease requires early diagnosis and risk stratification [1]. Heart failure (HF) is one of the most common CVD-related causes of death worldwide, impacting millions of people and placing a heavy strain on healthcare systems. HF is thought to be a pandemic that affects 64 million individuals globally. With an aging population, the prevalence of HF is anticipated to rise. According to the most recent estimates, there is projected to be a 46% increase in the prevalence of HF in the United States between 2012 and 2030 and a 12.7% increase in healthcare expenses [2].

HF is characterized by the heart’s reduced capacity to pump blood effectively, which leads to several incapacitating symptoms and complications [3]. Timely and accurate diagnosis is paramount for effective management and improved patient outcomes. Over the years, the identification and application of biomarkers has been fundamental in improving the diagnosis of HF. The concept of *biomarkers *was introduced in 1989 to identify measurable and quantifiable biological parameters to assess a patient’s health and physiology regarding disease risk and diagnosis [4]. WHO defines a biomarker as a substance or process that is measured objectively and can predict the occurrence or outcome of a disease [5]. For effective use, a biomarker must meet a range of criteria, including thorough assessment, cost-effectiveness, straightforward interpretation, and accessibility, while also being a significant pathophysiological mechanism in disease development [6].

Even though established biomarkers such as B-type natriuretic peptide (BNP) and N-terminal pro-B-type natriuretic peptide (NT-proBNP) have been valuable in clinical settings, there is a growing recognition of the potential offered by emerging biomarkers in further refining diagnostic precision [7]. The search for improved sensitivity, specificity, and a better comprehension of the complex nature of HF has led to the pursuit of novel biomarkers in this setting.

By reviewing recent research findings and available data, this study aims to explore the potential of emerging biomarkers in the early diagnosis of HF and the clinical value of early detection.

## Review

Classification of HF biomarkers

**Figure 1:**
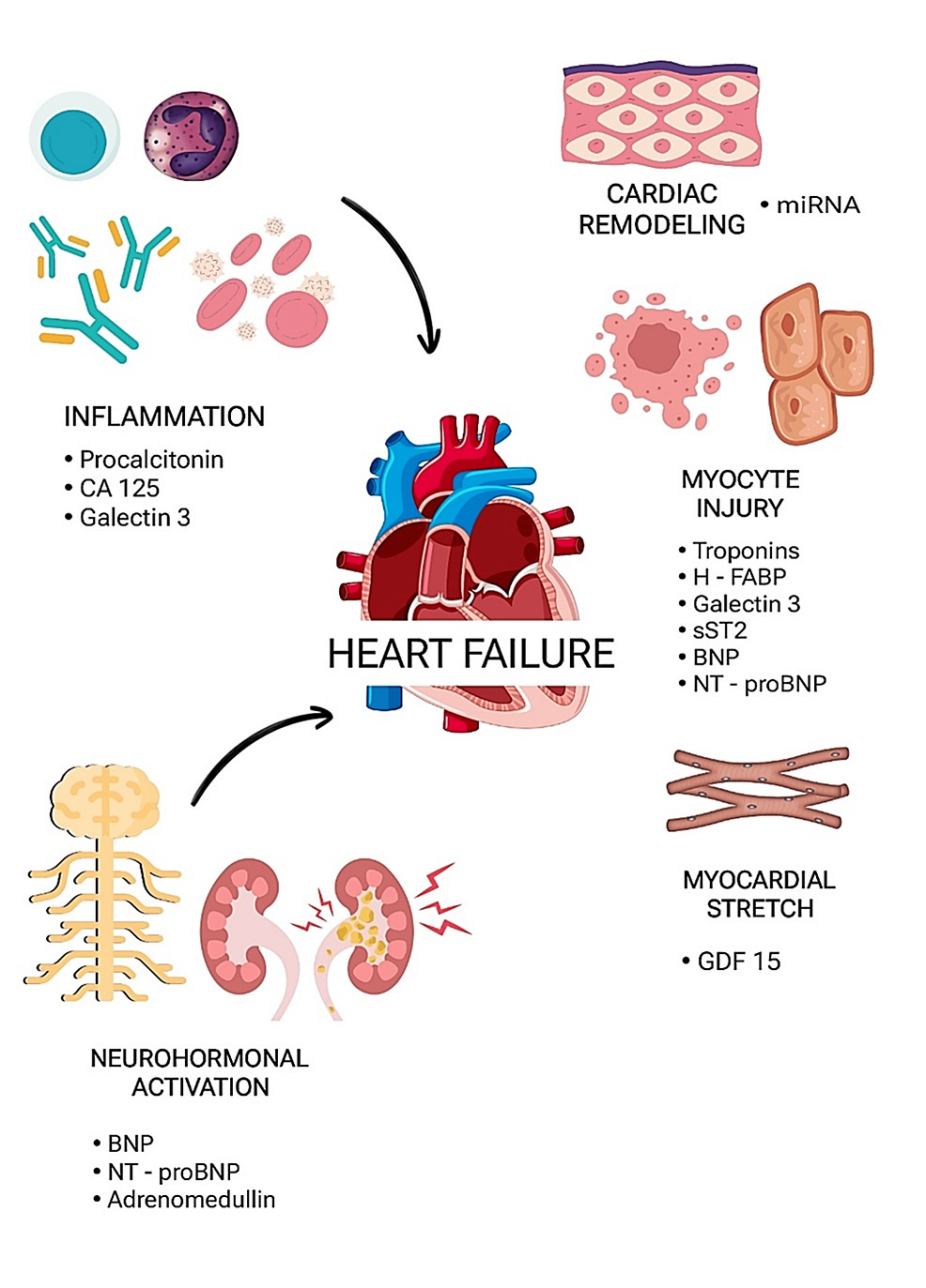
An illustration demonstrating the correlation between different biomarkers and the pathophysiological processes that underlie heart failure. The figure is created using www.Canva.com for graphic design. GDF-15 = growth differentiation factor 15; sST2 = soluble suppression of tumorigenicity 2; H-FABP = heart-type fatty acid-binding protein

Biomarkers offer a fast, low-risk, and inexpensive way to confirm or rule out the diagnosis of HF, assist in determining the prognosis, and, in addition, may yield significant information on the pathophysiology that outlines HF [8]. Based on their involvement in the pathophysiology of HF, biomarkers in HF can be broadly categorized (Figure 1). Numerous facets of the pathophysiology of HF continue to yield new indicators. In addition to potentially providing information on the etiology of the ailment, these biomarkers may also provide new treatment targets by reflecting disease processes at the entire body, organ, cell, or sub-cellular level [9].

Traditional cardiac biomarkers

Natriuretic Peptides

Natriuretic peptides (NPs) are crucial in preserving homeostasis in the cardiovascular system. They act as counter-regulatory hormones, helping to regulate volume and pressure overload [1]. NPs are currently indispensable in the diagnosis of HF, providing prognostic information and occasionally directing treatment [10]. Atrial natriuretic factor (ANP) was discovered in 1981 by injecting homogenized atrial tissues into rats, leading to decreased blood pressure and increased urine and sodium excretion [11]. Further study yielded more insights into the NP system, identifying BNP, also called brain-type natriuretic peptide, and C-type natriuretic peptide [10]. The ANP protein is encoded by the *NPPA *gene located on chromosome 1. It is translated and broken down into a 28 amino-acid bioactive form (ANP) and a 98 amino-acid N-terminal fragment (NT-proANP) upon stimulation and release. The half-life of ANP is around two minutes, but the half-life of NT-proANP varies depending on the specific fragment being evaluated [12]. ANP is primarily synthesized in the heart’s atria and, to a limited extent, in the ventricles and extracardiac tissues, among which is the kidney [13]. The main factor that results in the release of ANP is an increase in atrial wall stretch, which indicates an increase in intravascular volume. Additional factors that may result in release include catecholamines, arginine vasopressin, and endothelin [13]. The above factors demonstrate the counter-regulatory function of ANP against volume overload and hypertension.

Originally termed brain-type natriuretic peptide, BNP was first isolated from porcine brain tissues. Further studies revealed it is also produced in the cardiac ventricles [14]. Similar to ANP, BNP is a peptide neurohormone produced by cardiac ventricular myocytes due to mechanical stretching [15,16]. When the cardiac chambers are overloaded with excessive volume, stretching the cardiac muscle cells’ membranes triggers signal transduction. This process ultimately produces preproBNP, which undergoes translation and breakdown to produce the physiologically active C-terminal peptide, BNP, and the inactive N-terminal fragment called NT-proBNP [17]. BNP and NT-proBNP peptides are released in equal amounts into the bloodstream [18]. The serum half-life of BNP is 20 minutes, while that of NTproBNP is 120 minutes [19].

ANP increases kidney function by increasing blood flow, dilates afferent arterioles, and constricts efferent arterioles, leading to increased glomerular filtration [20]. It also hinders sodium reabsorption in the proximal tubule and inner medullary ducts, increasing urine production and sodium excretion [21,22]. ANP decreases blood pressure by reducing sympathetic output, increasing venous capacitance, and increasing vascular permeability [23]. ANP mediates these actions by inhibiting catecholamines, renin, angiotensin II, aldosterone, and endothelin [24]. An additional beneficial impact of ANP on the heart is the prevention of hypertrophy [12]. BNP induces increased intracellular cGMP signal cascading. These cascades reduce cardiac preload and afterload, which counteract the adverse effects of pressure and volume overload in HF [25]. ANP and BNP levels, therefore, indicate the severity of HF.

The bioactive form of ANP is unstable and has a short half-life, making precise measurement challenging [26]. The N-terminal prohormone fragment (NT-proANP) is more stable in blood but may have higher concentrations [27]. Several clinical tests, including immunoassays, have attempted to quantify NT-proANP, but it undergoes degradation. An assay focused on MR-proANP was developed in 2004. Recent studies have shown that even after being cleaved, preprohormone fragments can remain in their stable form. This implies that assays to quantify these fragments and explore their function as indicators of ANP physiology might be developed [27].

BNP and NT-proBNP in HF

The available evidence strongly supports the use of early serum BNP measurement to diagnose acute heart failure (AHF). The American Heart Association (AHA)/American College of Cardiology (ACC) guidelines for managing HF designate BNP measurement in all hospital admissions for AHF as a class I indication [28]. For patients who present with dyspnea in the emergency department (ED), cardiac biomarkers are beneficial because it can be challenging to distinguish between dyspnea brought on by pulmonary sickness and HF [10].

The study by Maisel et al. [28] was the first extensive investigation to assess the effectiveness of BNP as a cardiac biomarker for diagnosing HF in ED settings [28]. This study examined 1,586 individuals who presented to EDs with the primary symptom of dyspnea at seven different medical centers throughout the globe. Serum BNP levels were more significant in individuals with AHF-related dyspnea than in patients with non-cardiac dyspnea. Serum BNP levels were shown to be positively linked with the severity of HF. Serum BNP level was 90% sensitive and 76% specific for HF using a BNP threshold of 100 pg/mL. BNP exhibited a negative predictive value of 96% when using a 50 pg/mL threshold [29]. The PRIDE study, which investigated acute dyspnea in an ED, also reported similar findings when using NT-proBNP [30]. The NT-proBNP level was found to be highly sensitive and specific in diagnosing AHF among a group of 600 patients who presented to the ED with symptoms of dyspnea. A cutoff level of 300 pg/mL was used, and the NT-proBNP test showed a sensitivity of 90% and specificity of 85% for diagnosing AHF [30]. Due to the rapid and convenient measurement of serum biomarkers, using BNP in EDs can significantly decrease hospitalization duration and total expenses related to HF therapy [10]. In a study by Mueller et al. [30], 452 patients who came to the ED with acute dyspnea were assessed. The study revealed that using BNP monitoring resulted in a quicker diagnosis of HF, leading to shorter hospital stays and lower treatment expenses connected with ED visits [30].

Similarly, The IMPROVE-CHF study revealed the same results when using NT-proBNP. The measurement of serum NT-proBNP levels proved to be beneficial in diagnosing HF. It resulted in a 21% reduction in the duration of ED visits, a 45% decrease in the rate of rehospitalization after 60 days, and a corresponding reduction in the overall cost of treatment for these patients [31,32].

The biomarkers in the BACH study, which involved 1,641 patients experiencing acute dyspnea, found that MR-proANP was as effective as BNP and NT-proBNP in diagnosing AHF. Of the 568 patients diagnosed with AHF, MR-proANP had a sensitivity of 97.0% and a negative predictive value of 97.4% when the cutoff was set at 120 pmol/L [33].

Numerous studies have demonstrated that BNP levels are useful for prognosis in addition to their diagnostic value. Berger et al. [33] reported that elevated baseline serum BNP levels were more predictive of future sudden cardiac death in patients with a left ventricular ejection fraction below 35%. Sudden cardiac death was more prevalent among patients whose baseline serum BNP level was higher than 130 pg/mL, and it was suggested that patients with elevated BNP levels should undergo cardiac defibrillator therapy evaluation [33]. A Val-HeFT substudy found that patients with the highest percentage decrease in BNP from baseline had the lowest morbidity and mortality rates. In contrast, those with the highest percentage increase had the highest morbidity and mortality [34,35]. The REDHOT study assessed 464 patients who arrived at the ED with an initial BNP level over 100 pg/mL. The study discovered that individuals with baseline BNP levels over 200 pg/mL had a significant likelihood of experiencing adverse events within 90 days, including HF visits, hospitalizations, and mortality [36]. A subsequent investigation validated these results. The PRIDE study conducted a thorough examination of the one-year outcomes of patients who arrived at the ED with acute dyspnea [37]. Baseline NT-proBNP levels over 986 pg/mL were correlated with more severe HF. Furthermore, a single baseline NT-proBNP level beyond this threshold was the most influential individual indicator of mortality within one year [37]. Furthermore, in a meta-analysis, Doust et al. [37] found that in patients with both acute and chronic HF, a rise in blood BNP of 100 pg/mL was linked to a 35% increased risk of death [37].

MR-proANP has demonstrated substantial prognostic benefits for morbidity and mortality in acute and chronic HF [38]. Increasing levels of MR-proANP are associated with increased mortality in patients with AHF up to four years after presentation, according to multiple studies [39,40]. The results are comparable in chronic HF, where MR-proANP can predict mortality for over five years following the initial evaluation [41-43]. Several studies have demonstrated that serial monitoring of MR-proANP enhances mortality prediction in patients with chronic HF [44].

Serum NPs and HF Treatment Guidance

Multiple studies have demonstrated that implementing a BNP-guided treatment approach for outpatients with chronic HF leads to enhanced patient outcomes. The 2007 STARS-BNP trial evaluated the effectiveness of BNP-guided treatment strategies in improving HF outcomes. Of 220 patients, those receiving BNP-guided therapy had a significantly reduced primary outcome of HF-related mortality or readmission at 15 months [45]. The BATTLESCARRED study followed up the outcomes with NT-proBNP-guided clinical management in 364 HF exacerbated patients. Compared to standard care, a lower mortality rate in one-year follow-up was observed in the NT-proBNP-guided therapy group [46].

MR-proANP has not undergone the same level of investigation as BNP or NT-proBNP in terms of its potential for directing treatment in HF or other disorders. Further research is required to determine whether MR-proANP can replicate or improve upon the results achieved by BNP and NT-proBNP in directing therapy for HF.

Troponins

Troponins are structural proteins present in the thin filaments of the heart and skeletal muscles. The three components of the troponin complex I, T, and C, as well as calcium ions, are crucial for controlling muscle contraction. Cardiac T and I are primarily found in the heart, but troponin C is synthesized in skeletal and cardiac muscles [47]. Troponins are released upon myocardial injury, such as in acute myocardial infarction or pulmonary embolism [48]. Multiple generations of assays for cardiac troponin T and I have improved the sensitivity to identify cTn at lower concentrations. Troponin can be detected at the nanogram and picogram levels using the most recent generation of high-sensitivity troponin assays (hsTns). These assays can identify cTn in >95% of healthy subjects and at least in >50% of them [49]. HF is also associated with elevated troponin levels. Other causes of this rise include myocardial apoptosis, coronary ischemia, supply-demand mismatch with subendocardial ischemia, direct neurohormone toxicity, and inflammation [50]. Studies have shown that a positive cardiac troponin result is associated with higher hospital mortality, increased hospital stay, and higher chances of rehospitalization. Repeated measurements of cardiac troponins have prognostic value, and patients with a positive cTn test result tend to have lower systolic blood pressure on admission and a lower ejection fraction [51-53].

However, troponins are not necessarily disease-specific as they can be elevated in myocardial ischemia and chronic myocardial damage in HF. hsTns have increased the sensitivity to detect acute coronary syndromes but come with challenges. Increased cardiac troponins can be associated with both coronary and non-coronary heart diseases, making it non-specific to etiology and leading to false-positive interpretations. Additionally, troponins exhibit biological variability secondary to circadian rhythm and monthly and seasonal changes [54].

Troponins in HF

Several studies have investigated the role of troponin in diagnosing HF. More significant numbers of HF patients are found to have detectable cTn, and there is a constant correlation between the amount of cTn and the outcome of HF when new, more sensitive cTn assays are produced [55].

In addition to its diagnostic value, troponin has been recognized as a prognostic marker in HF. Contemporary studies consistently show an association between elevated troponin levels and adverse clinical outcomes. Arenja et al. [55] studied 667 patients who had acute dyspnea at presentation. Patients with AHF had higher levels of cTnI than patients whose acute dyspnea had non-cardiac etiology. After controlling for other risk variables, BNP and cardiac troponin I are potent predictors of short- and long-term prognosis in AHF patients and help reclassify patients based on mortality risk [55]. In hospitalized patients with decompensated HF, elevated troponin levels were an independent predictor of increased short-, intermediate-, and long-term mortality [56,57]. Demir et al. [57] conducted a three-year follow-up study among patients with HF. The study showed that the long-term mortality and morbidity rate in patients with CHF can be predicted independently by positive cTnT. Those with elevated serum cTnT levels may be able to identify patients with deteriorating CHF at an earlier stage [57,58].

Cardiac troponin levels can be elevated in HF, with significant prognostic value. Elevated levels in acute decompensated HF correlate with increased mortality and readmission rates involving both I and T isoforms of troponin [49]. The emerging evidence of the prognostic value of troponin has led to its utilization in guiding HF management. Treatment strategies targeting the reduction of troponin levels have shown promise in improving patient outcomes. Studies by Felker et al. [58] and Colombo et al. [59] focused on using troponin-guided therapy to optimize medical treatment and reported favorable effects on reducing HF-related hospitalizations and improving patient quality of life. Diagnosis and risk stratification of HF remains challenging due to its varied etiology and the absence of a definitive diagnostic test. However, troponin has emerged with promising value in diagnosing HF and predicting outcomes.

Novel cardiac biomarkers

Galectin-3

Within the lectin family, galectin-3 (Gal-3) is a β-galactoside-binding protein that regulates various physiological cellular processes, including apoptosis, differentiation, cellular adhesion, growth, and tissue repair. It is expressed in the extracellular space, mitochondrion, cytoplasm, nucleus, and cell surface [60,61]. Gal-3 can be a sensitive diagnostic or prognostic biomarker for various clinical disorders as it is quickly released to the cell surface and into fluids (including serum and urine) from damaged and inflamed cells [62]. The role of Gal-3 has been confirmed as a biomarker of fibrosis. According to numerous recent studies, Gal-3 is a novel heart disease biomarker. Clinical research suggests that serum and myocardial Gal-3 levels in HF may be valuable biomarkers for cardiac inflammation and fibrosis [63]. Ventricular remodeling of HF is closely associated with myocardial fibrosis and inflammation. Gal-3 plays a significant role in cardiac ventricular remodeling, contributing to its involvement in the pathogenesis of HF [64]. Gal-3 expression is usually low, but its synthesis and secretion increase with HF. The cardiac matrix, fibroblasts, and macrophages are the primary locations of Gal-3-binding sites. Gal-3 is secreted into the extracellular space at the site of damage and plays a crucial role in fibroblast activation [65].

Potential of Gal-3 as a Biomarker in HF

A case-control study was conducted by Khadeja Bi et al. [65] to evaluate the levels of Gal-3 in chronic HF patients. Gal-3 aids in the identification of chronic HF brought on by abnormal cardiac remodeling. The study concluded that measuring Gal-3 plasma levels can aid in diagnosing congestive HF with a 71% specificity and 92% sensitivity at the threshold level of 8 ng/mL [65,66].

An observational study was performed by Huang et al. [66], who monitored 223 HF patients. Patients with HF had significantly greater serum Gal-3 concentrations than the control group [66]. It was discovered that Gal-3 is a reliable indicator of HF. The sensitivity and specificity of Gal-3 as an HF diagnostic marker were shown to be 76.0% and 71.9%, respectively, at the threshold of 16 ng/mL [66,67].

Greater Gal-3 levels were associated with an increase in HF risk and mortality in patients with ambulatory HF. This finding suggests that Gal-3 is a potential indicator of long-term outcomes [68].

Serum Gal-3 exhibited a predictive value in both all-cause death and cardiovascular death in chronic HF, indicating its potential as a marker for identifying patients at increased risk of adverse outcomes [69].

Procalcitonin

Procalcitonin (PCT) is a calcitonin-derived propeptide. It is composed of 116 residues of amino acids and has a half-life of 24 hours. A very sensitive biomarker of inflammation, PCT has been employed for prognostic and diagnostic purposes in various conditions [70]. The correlation between inflammation and HF has been established. Stout et al. [70] proposed that mesenteric congestion may be the source of inflammation in AHF by facilitating bacterial translocation from the intestinal lumen into the bloodstream, resulting in endotoxemia and subsequent immune activation [70]. It is widely accepted that infections cause an increase in PCT. Patients with HF are prone to morbidity and mortality due to infections. With three-month readmission rates as high as 34%, HF patients are among the most commonly hospitalized. Davis et al. found that infections were the most common primary diagnosis for 30-day non-HF readmissions in a study involving patients with a first-discharge diagnosis of HF [71,72].

Role of Procalcitonin in HF

Patients with HF have been observed to have elevated PCT concentrations. Niebauer et al. [72] conducted the first study of PCT and demonstrated an increase in endotoxin and inflammatory cytokine levels during the edematous state of AHF [72]. When comparing patients with edematous HF to those with compensated HF as well as to controls without HF, PCT levels were found to be greater in the former group [73]. According to Sinning et al. [73], the PCT level was a reliable indicator of cardiovascular death, indicating that ischemia-induced cardiomyocyte damage raised PCT levels [73]. Villanueva et al. [74] found that elevated PCT levels were associated with higher mortality rates for AHF patients, regardless of white blood cell count, C-reactive protein, endotoxin, or interleukin (IL) levels [74]. PCT exhibited a substantial but moderate predictive value in AHF patients without clinical symptoms of infection at admission, according to a trial by Loncar et al. [75]. PROTECT, a multicenter, randomized, placebo-controlled trial, assessed the prevalence and clinical outcomes in patients hospitalized for AHF but did not show indications of active infection. It was shown that patients with raised PCT levels had considerably poor in-hospital and post-discharge outcomes [76,77]. The BACH experiment, a multicenter diagnostic biomarker research in ED patients with acute dyspnea, was the subject of a sub-analysis reported by Maisel et al. This analysis assessed the diagnostic value of PCT in patients with AHF. In the absence of antibiotic treatment, patients with PCT values >0.21 ng/mL had noticeably decreased chances of survival. If given insufficient antibiotics, AHF patients with low PCT values (<0.05 ng/mL) had a significantly increased mortality rate [78].

In a retrospective case-control study by Canbay et al. [78], there was no end-organ damage or active infection among HF patients. PCT results showed that 87.5% of HF patients were positive, and all controls were negative, with a cutoff level of 0.09 ng/mL. The corresponding sensitivity and specificity were 88.9 and 100% for this cutoff, respectively [78]. Additionally, PCT levels in inpatients and outpatients were analyzed. Inpatients had considerably higher concentrations of PCT. Serum PCT levels had a sensitivity of 84.2% and a specificity of 81.0% in distinguishing inpatients from outpatients [78,79].

Role of Carbohydrate Antigen 125 in HF

Carbohydrate antigen 125 (CA-125) is a glycoprotein released from a tissue present in the coelomic epithelium in the pleura, peritoneum, and pericardium [80]. On average, CA-125 has a half-life of five days [81]. The cellular activity of CA-125 is uncertain. It has been proposed that CA-125 shields the epithelial surface from mechanical stress by serving as a lubricant. CA-125 is usually elevated in cancers such as ovarian cancer. CA-125 could be a secondary cytokine enhanced in proinflammatory conditions due to primary cytokines such as IL-14 and tumor necrosis factor-alpha (TNF-α) [82]. Given the correlation between inflammation and HF, CA-125 can be a valuable biomarker in predicting the onset and severity of HF [83]. Although the exact mechanisms causing the upregulation of CA-125 in HF are not fully understood, both inflammatory and hemodynamic stimuli appear to be involved. The primary event in the production of CA-125 has been proposed to be the activation of mesothelial cells due to increasing hydrostatic pressure, mechanical stress, and cytokine activity [84].

Over the past 20 years, multiple analyses have verified the prognostic significance of CA-125 in HF. CA-125 has demonstrated a positive correlation with unfavorable clinical outcomes in patients with HF [85]. Yilmaz et al. [85] found that, in the sample of 150 patients with systolic dysfunction, there was a positive correlation between CA-125 and the probability of death and readmission [86]. To elaborate on the prognostic role of CA-125, Núñez et al. [86] performed a subanalysis of the BIOSTAT-CHF study. This multicenter cohort included 2,516 patients with HF [86]. Higher levels of CA-125 were positively correlated with the clinical congestion score, inflammatory mediators (IL-6 and GDF-15), and congestion indicators (NT-proBNP and bio-adrenomedullin).

Moreover, it was observed that elevated CA-125 levels were linked to an increased probability of one-year mortality risk and readmissions with HF. The degree of systemic congestion had no bearing on this predictive outcome [87]. In another study, Núñez et al. [87] assessed 529 individuals with acute HF in another cohort study. Apart from the conventional clinical data, the level of CA-125 was evaluated. Up to the six-month follow-up, serum levels of CA-125 are an independent predictor of mortality [87]. The predictive value of CA-125 in HF was investigated in a meta-analysis which included 16 studies with a total of 8,401 patients with AHF. Increases in all-cause mortality of 68% and readmissions owing to HF of 77% were found to be correlated with high CA-125 levels [88,89].

CA-125 as a Diagnostic Tool in HF

Currently, the most prevalent application of CA-125 is for diagnosing and monitoring treatment responses in ovarian tumors. However, many other malignant and non-malignant illnesses have also been routinely demonstrated to exhibit increased levels of CA-125, particularly when a serosal fluid component is involved [90]. Patients with HF have higher levels of CA-125 for a variety of causes; nonetheless, CA-125 lacks specificity and, despite having a comparatively high sensitivity, it is not a viable tool for screening or independent diagnosis of HF.

Adrenomedullin and Its Role in HF

Adrenomedullin is a neurohormonal peptide initially discovered in pheochromocytoma-derived tissue in 1993 [91]. It is a 52 amino acid peptide encoded by the *ADM* gene located on chromosome 11 [92]. Post-translation, it yields pro-adrenomedullin (proADM), which is subsequently converted to biologically active adrenomedullin (bio-ADM) [93]. Different body organs, such as the kidneys, lungs, endocrine, and cardiovascular systems, generate proADM and bio-ADM in response to volume overload, which is frequently observed in HF. These increased levels of bio-ADM in HF indicate an attempt to limit the volume overload by stabilizing the endothelial barrier function [94].

Biomarkers that support accurate congestion assessment are desperately needed because congestion is infamously hard to measure accurately with high observer variability [95]. Recent studies highlight the strong association of increasing bio-ADM with systemic congestion and adverse outcomes independent of NT-proBNP [96]. Bio-ADM can be valuable for diagnosing HF and can be used to determine clinical outcomes. A study by Pandhi et al. [96] suggested that the pre-discharge values of bio-ADM in hospitalized patients with HF can help assess residual congestion, thereby recognizing patients at risk of readmission [96]. Apart from this, the study also showed that higher bio-ADM levels are associated with poor response to diuretic treatment and more extended hospital stays [97].

Adrenomedullin and Traditional Biomarkers

Kremer et al. [97] found that bio-ADM in patients with AHF is shown to decrease with appropriate decongestion and symptom improvement over seven days, in contrast to BNP, where they could not identify this response [97]. Another advantage of this over traditional biomarkers is its specificity to systemic vascular congestion, which may facilitate better targeting therapy for cardiac congestion when added to a multimodal clinical assessment strategy [98,99]. The relationship between bio-ADM and the benefit of diuretics post-discharge and residual tissue congestion appears more prominent than that of NT-proBNP, primarily representing elevated left ventricular filling pressures and intravascular volume overload [95]. A study by Egerstedt et al. [95] reported that bio-ADM elevated above 39 pg/mL conferred increased odds of HF diagnosis, incidence of dilated vessels, and pleural effusion on X-ray [95]. This study also revealed the association of bio-ADM with hospitalization and 30- and 90-day mortality independent of NT-proBNP. Bio-ADM values between the ranges of 46.2 of 96.5 pg/mL were said to be associated with increased incidence of hospitalization and adverse cardiac events [96].

In conclusion, bio-ADM has tremendous potential for evaluating prognosis and therapy response. Although it is a tool to assess treatment plans, avert unfavorable consequences, and avoid readmissions, its sensitivity and specificity as an HF diagnostic biomarker are yet to be established.

Role of Heart-Type Fatty Acid-Binding Protein as a Biomarker

Heart-type fatty acid-binding protein (H-FABP) is a low-molecular-weight (14-15 kD) transport protein that belongs to a group of cytoplasmic proteins known as fatty acid-binding proteins (FABPs) [100]. FABPs are present abundantly in tissues where fatty acid metabolism occurs, such as the heart, kidney, mammary glands, and brain [101]. Various texts state that h-FABP is released into circulation in response to myocardial injury. Unlike cardiac troponin, which is attached to the contractile components of cardiomyocytes, it is present in the cytoplasm of cardiomyocytes in a soluble form, making it detectable within two hours of myocardial damage [102,103]. In addition to reducing diagnostic delays, this speeds up the start of treatment plans and prevents further organ damage.

A study by Niizeki et al. [103] showed the independent association and increased sensitivity of H-FABP with ongoing myocardial damage, especially in chronic HF. It was noted that higher levels of H-FABP on admission were associated with mortality as well as non-fatal cardiac events [103]. The correlation of increasing levels of H-FABP in children with chronic HF depicts the severity of the condition [104]. Hoffman et al. [101] corroborated this by reporting that each unit increase of H-FABP by 10 ng/mL being measured only once in the ED was associated with a 2% increase in all-cause mortality and a 3-7% increase in related rehospitalization risk at five years [101,102].

Another study by Shirakabe et al. [105] among patients with worsening renal failure showed H-FABP as a more effective marker for predicting adverse long-term outcomes, especially in patients with HF as an inclusion criterion [105]. The potential role of H-FABP in aiding the treatment of HF was studied which showed decreasing levels of H-FABP in response to HF treatment by ivabradine. This decrease can be attributed to improved myocardial perfusion throughout the treatment [106,107].

H-FABP and Traditional Markers

H-FABP elevation can also be attributed to renal mechanisms apart from cardiac causes. Hence, one must also consider glomerular damage as one of the causes of raised H-FABP [108]. However, when combined with serum NT-proBNP and hsTnT, which are both specific to myocardial injury, H-FABP has high efficacy [106]. In a study by Hoffman et al. [101], among 401 study participants, there was an overall increase in sensitivity and specificity of combined biomarkers (NT-proBNP and H-FABP) when compared to that of NT-proBNP alone [101].

Although the prognostic potential of H-FABP outweighs its diagnostic role, there is scope for its use as a screening and rapid diagnostic tool that needs further evaluation.

MicroRNA

MicroRNAs (miRNAs) are crucial for gene expression in eukaryotic cells. They are transcribed by RNA polymerase II (Pol II) as primary miRNAs and converted into precursor miRNAs by the Drosha, RNase enzyme, and its cofactor. These pre-miRNAs undergo further cleavage by Dicer and its cofactors, transforming into mature, double-stranded miRNAs. It has been discovered that bodily fluids such as urine, saliva, and plasma contain miRNAs [109].

The formation of a normal, functioning heart depends on miRNAs, which are crucial regulators at different stages of the development of the heart. Thus, miRNAs play a significant role in cardiovascular physiology [110]. In animal models, numerous miRNAs have been linked to essential processes such as cardiac hypertrophy, fibrosis, and apoptosis. Rao et al. [109] performed a study in mice where they eliminated the gene essential for miRNA synthesis, dgcr8, specific to cardiomyocytes. The results showed a phenotype that exhibited left ventricular dysfunction and progressed to dilated cardiomyopathy and premature death [109]. Chen et al. [110] found that a cardiac-specific deletion of the gene Dicer, which codes for an RNase III endonuclease necessary for the processing of miRNA, causes rapidly progressing DCM, HF, and postnatal mortality. Mice with Dicer mutations exhibit severe sarcomere disorder and misexpression of heart contractile proteins. Dicer expression was also significantly increased in human hearts with failing and end-stage dilated cardiomyopathy hearts following the implantation of left ventricular assist devices to enhance cardiac function [111,112]. Karakikes et al. [111] conducted a trial on rats, which showed that miR‐1 is an essential modulator of cardiac hypertrophy. According to the study, restoring miR-1 gene expression may be a unique therapeutic approach to stop maladaptive cardiac remodeling and reverse pressure-induced ventricular hypertrophy [111]. A similar role of miRNAs was observed in human hearts when myocardial biopsies were studied from patients [112,113].

Association of miRNA with HF

MiRNAs are intriguing candidates for novel biomarkers in HF, given the evidence connecting them to the onset and progression of HF. Corsten et al. [112] examined the plasma levels of specific miRNAs in patients with acute myocardial infarction, viral myocarditis, diastolic dysfunction, and AHF in 2010. They found that miR-499 was significantly higher in HF patients [112]. Numerous miRNAs were studied in HF patients by Tijsen et al. [113]. In their findings, miR423-5p was highly correlated with the diagnosis of HF at hospital admission [113]. Although it showed promise as a diagnostic predictor of HF, these outcomes were attained in a small sample size. In another study by Bauters et al. [114], circulating levels of miR-133a and miR-423-5p did not correlate with NP levels. These miRNAs were ineffective as markers of left ventricular function and remodeling one year after myocardial infarction [114-116]. Yang et al. [115] reviewed four relevant studies regarding circulating miRNAs in patients with HF. The findings showed that low levels of several miRNAs, including miR-30, miR-423-5p, and miR-18, were associated with worse overall survival of patients, indicating their significant prognostic value in HF [115].

Patients with dyspnea who experience HF were distinguished from those who do not by levels of circulating miRNAs in a study. Compared to healthy individuals and those with other causes of dyspnea, patients with HF exhibit distinct expression levels of miR-423-5p. miRNA expression also varies in AHF, with high levels of miR-499 and low levels of miR-103, miR-142-3p, miR-30b, and miR-342-3p. MiRNAs can also differentiate between HF with reduced and intact ejection fractions [110].

A study involving 30 HF patients and 30 healthy controls found elevated miRs 423-5p, 320a, 22, and 92b in individuals with HF. These miRNAs were combined to create a predictive score for the diagnosis of HF. This multimarker approach showed 90% sensitivity and specificity in diagnosing HF. The score could be a non-invasive tool in assessing left ventricular structure and function [109].

GDF-15 and HF

GDF-15 regulates inflammatory pathways, apoptosis, cell repair, and growth, strongly linking it to cardiac hypertrophy and fibrosis [116-118]. Initially produced as a pro-GDF-15 dimer within the cytoplasm, GDF-15 undergoes cleavage and is released into the bloodstream as mature GDF-15, measuring 25 kDa [119].

While GDF-15 is typically highly expressed in the placenta and prostate and scarcely in other tissues, its expression increases in conditions such as myocardial ischemia, atherosclerosis, and cardiac pressure overload, playing a protective role in the myocardium [120]. GDF-15 expression is found in various cell types, such as cardiomyocytes, adipocytes, macrophages, endothelial cells, and vascular smooth muscle cells of healthy and diseased individuals [118].

Recent research identifies GDF-15 as a novel marker for detecting HF. This marker is associated with mitigating myocardial stress and assisting in ventricular remodeling. GDF-15 plays a dual role in ventricular remodeling: it can promote myocardial fibrosis and collagen protein deposition while inhibiting myocardial hypertrophy [121]. GDF-15 is strongly correlated with left ventricular remodeling (LVR) through echocardiography, suggesting its potential involvement in LVR [121].

Kempf et al. [120] studied the increasing level of GDF-15 in 455 patients with chronic HF. About 75% of the involved participants had GDF-15 levels above average, and increasing GDF-15 concentration was associated with increasing symptom severity of HF [120]. In advanced HF (New York Heart Association class III), five biomarkers, including PCT, NT-proBNP, GDF-15, Gal-3, and troponin, were assessed. GDF-15 emerged as the strongest predictor of long-term mortality, surpassing NT-proBNP in predictive value [121-123]. GDF-15 independently forecasted death or initial HF rehospitalization in both HF subtypes using crucial clinical predictors such as hsTn and NT-proBNP. In a study of 455 chronic HF patients (median follow-up: 40 months), GDF-15 predicted mortality regardless of clinical and laboratory variables [124]. Additionally, a Val-HeFT post hoc analysis revealed that changes in GDF-15 over one year remained a standalone predictor of death. Unlike NT-proBNP, GDF-15 remains unaffected by atrial fibrillation, suggesting its potential usefulness in these patients [124].

HF is a significant medical concern, often attributed to ischemic heart disease (IHD), the most prevalent cause of HF. A study by Elsewify et al. [123] aimed to compare plasma BNP levels, hsTnI, and serum GDF-15 between HF patients with and without IHD [123]. A serum GDF-15 cutoff of ≤717 pg/mL demonstrated the highest specificity at 85.51%, BNP at a cutoff of >264 pg/mL at 50.72%, and 59.42% for hsTnI. At a cutoff of >45.2 pg/mL, hsTnI exhibited the highest sensitivity, 70.59%, 68.63% for BNP, and 33.33% for GDF-15 in distinguishing HF with IHD from HF without IHD [123,125].

GDF-15 is promising as a new and effective biomarker for diagnosing HF, long-term mortality, and HF hospitalization due to its high diagnostic performance. However, more significant, high-quality prospective investigations are needed to validate its accuracy and consistency.

sST2

Within the IL-1 receptor family, ST2 exists in two isoforms: membrane-bound (ST2L) and soluble (sST2). In addition to an intracellular SIR domain similar to TLRs and other IL-1Rs, ST2L possesses three external IgG domains and one transmembrane domain. The intracellular and transmembrane domains are absent from the soluble ST2 isoform (sST2) [124-126]. sST2 is a novel biomarker and has been added to the 2013 ACCF/AHA guideline to risk stratify patients with HF [127].

Patients with HF have higher sST2 levels, and it has been suggested that the lungs play a significant role in the creation of sST2 when HF is present [128]. In response to fibrotic and inflammatory stimuli, cardiac fibroblasts and myocytes also create sST2 [129].

IL-33 is an IL-1-like cytokine released by stromal cells in cardiac and extracardiac tissues in response to cell injury [130]. By attaching to the transmembrane receptor ST2L isoform, IL-33 produces beneficial effects such as lowering myocardial fibrosis and cardiomyocyte hypertrophy in response to angiotensin 11 or phenylephrine and apoptosis by inducing antiapoptotic factors and suppression of caspase three activities [131]. High sST2 concentrations thus increase apoptosis, cardiomyocyte hypertrophy, and cardiac fibrosis, which eventually cause irreversible damage after myocardial infarction, leading to HF. This also signifies a worse prognosis in patients affected with HF [131,132].

A concentration of sST2 greater than 35 ng/mL was established as a positive cutoff value for identifying individuals with HF who are at high risk [133]. Research has also demonstrated that as sST2 is less affected by renal function than BNP, it can be utilized in conjunction with BNP as an additional diagnostic marker for HF [134]. NTPro-BNP and sST2 both exhibited a high diagnostic accuracy for HF in a single-center study, but sST2 had an even better prediction capacity for fatal outcomes, including in-hospital and one-month mortality rates [130]. The function of sST2 in characterizing AHF in individuals presenting with acute dyspnea was examined in the PRIDE trial. sST2 was judged to be more significant as a prognostic tool for predicting death from HF, even though NT-proBNP was found to be significantly better than sST2 for diagnosing AHF. Furthermore, the highest death rates after one year and four years, reaching approximately 40%, were linked to the combined elevation of sST2 and NT-proBNP. The study suggested that a threshold of sST2 ≥35 ng/mL could be used to predict a poor prognosis and risk of death [135].

Yamamoto et al. [134] assessed the added clinical value of sST2, pentraxin 3, Gal-3, and hs-TnT beyond BNP for risk stratification in a sizable Asian population. They found that sST2 was linked to significant outcomes (hospitalization for HF, cardiovascular mortality, and all-cause mortality) in patients suffering from acute decompensated HF only in those with preserved ejection fraction [134]. Greater levels of sST2 were strongly connected with a greater rate of poor outcomes (heart transplantation or all-cause mortality) [136,137]. Furthermore, several multicenter studies, such as the ASCEND-HF trial and the MOCA study, demonstrated that sST2 concentration had a strong ability to predict cardiovascular mortality over the long term (30 days) as well as the short term (one year) [138].

Thus, in conclusion, both upon admission and at the scheduled discharge in cases of AHF, sST2 can be monitored. A more extended hospital stay, a quicker up-titration of HF medications (after hemodynamic stabilization), more frequent follow-up visits following discharge, or monitoring devices to identify pulmonary congestion may be indicated for patients whose sST2 levels do not decline. sST2 readings in patients with chronic HF predict outcome and reverse remodeling in patients. Therefore, sSt2 assessment is a valuable tool for risk stratification, either by itself or in conjunction with troponins and natriuretic peptides.

Table 1 depicts a summary of cardiac biomarkers and their diagnostic and prognostic factors.

**Table 1:** A summary of the cardiac biomarkers and their diagnostic and prognostic potential in heart failure. BNP = brain natriuretic peptide; NT-proBNP = N-terminal pro-B-type natriuretic peptide; CA-125 = carbohydrate antigen 125; AHF = acute heart failure; H-FABP = heart-type fatty acid-binding protein; CVS = cardiovascular; GDF-15 = growth differentiation factor 15; sST2 = soluble suppression of tumorigenicity

Biomarkers	HF Prognosis	Advantages	HF diagnosis
BNP	+	High negative predictive value [124]. Improves survival and hospital stay [139]. Easily accessible diagnostic methods [140]	+ At a threshold of 100 pg/mL [29], sensitivity of 90% and specificity of 76%
NT-proBNP			+ At a cutoff level of 300 pg/mL [30], sensitivity of 90% and specificity of 85%
Troponins	+	Risk stratification and powerful predictor of mortality at 3 months [141]. Easy to access [142]	+
Galectin-3	+	Predictive of long-term outcomes and mortality [68]	+ At a threshold of 16 ng/mL [67], sensitivity of 76.0% and specificity of 71.9%. At a threshold level of 8 ng/mL [66], sensitivity of 92% and specificity of 71%
Procalcitonin	+	High positive predictive value [79]. Assessment of severity	+ At a cutoff level of 0.09 ng/mL [79], sensitivity of 99.9% and specificity of 100%
CA-125	+	Treatment response. Independent predictor of mortality in 6 months of follow-up [88]. Easily available in clinical labs. Standardized measurement [85]	+ For diagnostic sensitivity [89]. Limited specificity
Adrenomedullin	+	74% accuracy in predicting 90-day survival with AHF [143]. Therapy guidance [99]. Independent predictor of hospitalization and mortality [96]	+ [96] Limited evidence supporting sensitivity and specificity
H-FABP	+	Independent predictor of all-cause deaths and CVS-related deaths [102]	+ [144] Limited evidence supporting sensitivity and specificity
miRNAs	+	Differentiates among individuals with no-HF, HFpEF, and HfrEF [110] Predictors for HF hospitalization and all-cause mortality [117]	+ Combined miRs 423-5p, 320a, 22, and 92b [109]. Sensitivity of 90% and specificity of 90%
GDF-15	+	Variations in levels over one year strongly predict mortality [124]	+ [145] Limited evidence supporting sensitivity and specificity
sST2	+	Risk stratification [127]. Predictive of all-cause deaths and CVS-related deaths [135]	+ [133] Limited evidence supporting sensitivity and specificity

Multiple-marker approach

Cardiac failure encompasses a sequence of abnormalities culminating into a symptomatic syndrome, with each biomarker indicating an underlying pathology. Relying on a single marker to detect a complex multifactorial pathology may underestimate the risks involved [146]. The multiple-marker method involves quantifying multiple biomarkers in the blood or other bodily fluids at the same time. The multiple-marker approach assesses many biomarkers associated with the intricacy of HF to improve risk assessment and diagnosis precision [147]. This technique has improved risk prediction and overall morbidity and mortality compared to standard risk assessment approaches by quantifying a single biomarker [148].

Multiple markers help curtail the individual limitations of each biomarker. For instance, NPs are elevated in left ventricular overload. However, they may also be elevated in ischemia [149]. Multiple markers can potentially be more precise and efficient biomarkers of diagnosis, prognosis, and therapy response when paired with additional biomarkers indicative of the multiple pathogenic processes of HF [150]. Risk of mortality is a crucial indicator for long-term survival in cardiac failure, and a recent study examining NT-proBNP and ST2 levels in AHF revealed that the combined elevation of both biomarkers significantly increased the risk of mortality compared to the elevation of a single marker [151].

The Framingham Heart Study found that individuals with the highest quartile scores for sST2, GDF-15, and hs-TnI multimarkers had six times higher risk of HF and three times higher risk of death over an 11-year follow-up. However, when the score was combined with clinical variables, there was a significant net reclassification improvement [152].

Cardiac failure is a complex multifactorial condition influenced by various pathological factors, making the longstanding desire to find a single biomarker indicative of its risk highly challenging. An ideal biomarker should reflect the underlying pathophysiology causing HF; be measurable within a brief time frame for clinical significance; furnish information on risk, prognosis, and the necessary therapeutic interventions; and be detected by widely comprehensive and standardized methods [153,154]. However, most biomarkers need to meet each of these requirements. Consequently, relying on a single marker for every patient is not practical.

Every marker signifies a distinct pathophysiological condition that has malfunctioned in cardiac Failure. The method and time to study the biomarker vary [155]. Measurement of biomarker levels exhibits variability across machines and hospitals. The development of point-of-care devices offers precise and rapid detection of biomarker levels. However, not all institutions can adopt such commercialized products due to affordability constraints, limiting access for some institutions [156]. Patient factors also limit the use of biomarkers. One contributing factor is the difference in the distribution of biomarker levels between individuals with and without cardiovascular events. For example, H-FABP may be higher in those with increased skeletal mass [157].

A study by Melander et al. [156] found that incorporating novel biomarkers facilitated participants’ reclassification into high-, intermediate-, and low-risk categories, surpassing the previous determination based solely on cardiovascular risk factors [9]. While the statistical significance of this approach is well proven at p < 0.001 for N-BNP and CRP, the p-value for the increase in Net Classification Index remained relatively non-significant at p < 0.52. Clinical significance is crucial to guide participants to various preventive and therapeutic approaches. Due to this, people who were previously deemed to be at high risk due to cardiovascular risk factors such as obesity, comorbidities, food, and smoking habits may be falsely classified as low risk due to the level of biomarkers, giving the appearance that they are healthier than expected [156].

A recent discovery of genetic biomarkers that code for the cardiac troponin T protein enables a customized evaluation of relative risk [158,159]. However, challenges arise as many variants are situated in non-coding regions, hindering the identification of their function or targets. Moreover, most loci impact multiple genes and phenotypes, complicating the causal variant identification [159]. Beyond heritability, cardiac disease is primarily influenced by environmental and lifestyle factors. The laboratory methodology for genetic biomarkers is time-consuming and expensive for clinical routine compared to other biomarkers. These genetic biomarkers do not add significant additive value to classic cardiovascular risk factors [159].

Accuracy and reliability are to be looked into when developing a new marker. The optimal study design for directly assessing the impact of a biomarker is a well-designed randomized controlled trial [160]. New tests in the development of biomarkers should undergo evaluation across a diverse range of individuals representative of the typical diagnostic scenario, and the statistical methods employed for evaluating the clinical significance of biomarkers should be standardized. Hence, this necessitates a phased approach [161]. Due to the significant costs and time associated with conducting phased randomized trials, it is impractical to execute a comprehensive systematic trial for all biomarkers [162].

Hence, the biomarkers that are cost-effective and perform well in rapid testing to rule out disease, thereby helping in the quick analysis and allowing quick appropriate therapy, will be more prevalently used compared to those that require further trials to improve efficacy and minimize side effects and survive the competition [150]. Crucial to achieving clinical acceptance of a proposed biomarker is laboratory and translational research that establishes a plausible pathological basis [150].

## Conclusions

Novel biomarkers have been shown to have higher sensitivity and specificity compared to traditional biomarkers. This allows for a more accurate and timely diagnosis of HF, thus allowing for timely interventions. Novel biomarkers have also been found to have better prognostic abilities and better prediction for fatal outcomes. Some are also helpful in differentiating between cardiac and non-cardiac causes of dyspnea. They can be used for risk stratification, and by regularly monitoring them throughout treatment, adjustments to treatment plans can be made.
